# The impact of green innovation initiatives on competitiveness and financial performance of the land transport industry

**DOI:** 10.1016/j.heliyon.2023.e19130

**Published:** 2023-08-15

**Authors:** Josephine D. German, Anak Agung Ngurah Perwira Redi, Ardvin Kester S. Ong, Jerome L. Liwanag

**Affiliations:** a School of Industrial Engineering and Engineering Management, Mapúa University, Manila, Philippines; b Industrial Engineering Department, Sampoerna University, Jakarta, Indonesia; c E.T. Yuchengco School of Business, Mapua University, Makati, Philippines

**Keywords:** Green innovation initiatives, Sustainability, Transportation, Firm competitiveness, Financial performance

## Abstract

The transportation sector is one of the primary contributors to greenhouse gas emissions that have deteriorating effects on the state of the environment. The implementation of sustainable practices has become one of the most challenging tasks of organizations at present. This study examined the effect of implementing green innovation initiatives on a firm's competitiveness and financial performance of motor vehicle companies in the Philippines. Data were gathered through an online survey questionnaire with a total of 206 respondents composed of employees of various ranks working in companies engaged in the manufacture, distribution, retail, and service of motor vehicles. The theoretical framework presented a hierarchical latent variable model which was validated using the partial least square structural equation modelling (PLS-SEM). The model fit, measurement, general construct fit, discriminant validity, and structural model parameters were examined and found to have acceptable values. The findings indicated that environmental regulations, market demand, government pressure, competitor pressure, corporate social responsibility, and employee conduct were the significant drivers of green innovation initiatives. The study also revealed that the implementation of green innovation initiatives positively affects the firm's competitiveness and financial performance. Motor vehicle companies and other types of organizations are encouraged to demonstrate not only their concern for society or community but also their concern for the environment to acquire better market leverage and financial position.

## 1 Introduction

Logistics, as a significant function in the supply chain, plays a crucial role in the success of many businesses. Firms utilize logistics to increase their competitiveness by attempting to deliver the correct product at the right time, location, and to the appropriate consumer. Logistics is a lever for improving the articulation of the productive sectors, and hence the economic development that enhances citizens' quality of life from the public sector's standpoint [1]. Materials, capital, and information flows are part of supply chains, which are enterprise groups with varying degrees of autonomy, such as suppliers, manufacturers, and sellers [2]. Supply chains are networks of manufacturers, suppliers, transporters, warehouses, and retailers that transform and distribute products or services to customers [3].

The COVID-19 pandemic greatly impacted finished goods delivery as changes and problems in the transportation sector happened worldwide. The transportation sector has decreased drastically everywhere due to restrictions imposed in various countries and territories [4]. Yang et al. [5] indicated that in the early phase of the pandemic, the increased transportation requirements for medical supplies and people's daily necessities put a lot of strain on the logistics systems. Further effects of the pandemic were raising protection costs, lowering output quality, and reducing transportation demand in families and the manufacturing sector [6]. Supply chain adaptation is inevitable to achieve functional recovery, requiring visibility and communication with suppliers for early detection of potential shutdowns and inventory-increasing steps [7].

Community quarantine was one of the adaptation measures made by the Philippine government to reduce local transmission of COVID-19 in the country [8]. This measure includes restrictions on the mobility of individuals, large groups of people, or whole communities in a particular area, encouraging people to stay and work at home. Public utility vehicles (PUV) are considered the predominant mode of transportation in the country [9], and the prohibition of PUV operations significantly reduced carbon emissions during the quarantine period. Over the past years, individuals have become accustomed to the situation and adjusted their way of living and working. Home delivery has become a trend that led to the rise of e-commerce [10], increasing the number of vehicles utilized in this sector. According to the Land Transportation Office, the transportation agency in the Philippines, vehicle registration increased by 9.88% from 2020 to 2021 [11]. One of the primary producers of industrial waste that degrades the quality of the environment is the automotive industry [12], and shipping emissions contribute significantly to particulate matter (PM) and nitrogen dioxide (NO2) levels [13,14]. The rising population of people and transportation also requires immediate consideration of implementing sustainable initiatives in the business sector.

Green innovation and other environmental management techniques are crucial for cultivating fruitful stakeholder partnerships [15]. Organizations are keen on implementing green innovation initiatives (GII), a unique tradition that minimizes the emission of hazardous pollutants throughout the entire life cycle and the consumption of natural resources [16]. GII considers green innovation practices in the product, process, and management [17,18]. According to Suhaily et al. [19], green product innovation refers to developing goods that could protect or enhance the environment, conserve energy and resources, and reduce the use of harmful substances, pollution, and waste. It includes the choice of materials, energy use, and pollution prevention throughout the product life cycle [20]. Green process innovation refers to designing and manufacturing a product to improve resources and energy usage [21,22] and reduce waste [18] during operation. Lastly, green management innovation denotes creating and adopting a new management technique within the organization [23], including environmental management, energy management, and quality management [24]. Businesses currently engage in both exploratory and exploitative green technologies to address environmental concerns by advancing existing capabilities that can give them a competitive edge for a successful operation [25].

Several works of literature have discussed adopting and implementing green initiatives in the transportation industry to protect the environment. Li and Li [26] recognized that green technology innovation is critical for lowering carbon emissions and achieving long-term development in China's transportation sector. Using the Hausman test, Robustness test, and Fixed Effect (FE) model, they found that green technology innovations of transportation companies have a positive role in promoting South-South cooperation (SSC) development. Transport companies realized that boosting green technical innovation by one percent led to a 0.23% drop in carbon emissions and helped improve market competition and social networks. In another study, Zailani et al. [12] identified that environmental regulations, market demand, and the firm's internal initiatives are the key factors that affect a firm's GII, which leads to improvement in the ecological, social, and economic performance of the Malaysian automotive supply chain. Concerning green product innovation, Lin et al. [27] focused on the Vietnamese motorcycle industry and looked at how market demand influences green product innovation and firm performance. Their findings indicate that market demand positively influences environmental performance, economic performance, and products, while environmental performance, products, financial performance, and market demand significantly affect firm performance.

Recent studies have shown how development and growth have focused more on green technologies such as water, transportation, health, and energy. Taking the study of Wang et al. [28], they considered the evaluation of green technology development from 1990 to 2015. Their review study presented how the green technology, especially in health and vehicle made great achievements, even in climate mitigation. However, their study explicitly demonstrated challenges in the near future to fully rely on green technology due to several limitations of a country. Using time series econometric techniques, Sun et al. [29] explained that tourism frown on large carbon footprint and emissions. This was suggested to be the focus of development on eco-innovation. Razzaq et al. [30] presented the limitations of green technology and innovation on the aspect of transportation infrastructure. Using nonlinear autoregressive distributed lag analysis of prior dataset, they found that transport infrastructures are limited and have difficulty in development due to constraint in economic aspects. On a positive note, Chu et al. [31] showed how an increase in green technology advancement in the field of transportation also increases financial performance. Using a Likert-scale survey with hierarchical-moderated regression analysis, their findings highlighted the need for third-party logistics to focus on green innovation initiatives to better promote the company's financial interests. As such, Irfan et al. [32] explained how green financial efforts could uplift green innovation initiatives among companies. Using mediation effect, difference-in-difference, and autoregression, their study solidified how green innovation initiatives can promote financial performance, sustainability performance, even in different parameter testing. Consistent results have justified the need for further involvement and exploration of GII in the transportation sector.

In the manufacturing sector, Irfan et al. [33] recognized that resource constraints and environmental concerns had made asset sustainability and pollution one of the most pressing worldwide issues. Using PLS-SEM, they investigated the factors that affect environmental and organizational performance and the moderating role of innovation orientation in Pakistan's manufacturing and service firms. Results indicate that competitive pressure, government pressure, and employee conduct positively and significantly impact green innovation practices. In another study, Acquah et al. [34] investigated how total quality management (TQM) mediated in the linkages between green procurement, green product innovation, and green process innovation to promote green organizational legitimacy and access to green finance. They used PLS-SEM to analyze the data, and the results showed that green product and process innovation improved TQM, green organizational legitimacy, and access to green financing. At the same time, TQM had varying degrees of mediating power over the effects of green procurement, green product innovation, and green process innovation on green financing. Huang et al. [35] also explored the impact of regulatory and customer pressures on green innovation performance through green organizational responses in manufacturing organizations in central China. Their findings show that regulatory and customer pressure increases green corporate responses and improves green innovation performance. Although customer pressure strongly influences research and development investments and collaboration networks, regulatory pressure has no meaningful impact on these aspects.

Management innovation is also a recognized topic for research. Business trends that have changed quickly due to the competitive environment were examined by Kraus et al. [36]. Using the PLS-SEM, they evaluated the influence of corporate social responsibility (CSR) on the environmental performance of large industrial enterprises in Malaysia. Results show that while CSR does not directly affect environmental performance, it is positively connected with environmental strategy and green innovation. Environmental performance was also positively correlated with environmental strategy and green innovation. In another study, Sellitto et al. [37], realized how green innovation helped a cluster of furniture enterprises in Southern Brazil gain a competitive advantage. Their research provided a framework of manifest variables that link industry business strategies, competitive enablers, and green innovation. They found that eco-efficiency methods used in green innovation have a beneficial influence on the competitive enablers and the competitive advantage.

Various external factors can influence a firm's competitiveness. Raza [38] noted external institutional drivers such as environmental regulations, adoption of green innovations, and the impact of such innovations affect a firm's environmental and economic performance. Findings revealed that regulatory pressure had a substantial positive effect on adopting green technology and process improvement. Similarly, green technical innovation had a considerable positive impact on environmental performance and substantially influenced a firm's economic performance. In China, Peng et al. [39] discovered that managing and incentivizing environmental regulation significantly impact a firm's intention to engage in green innovation.

Investigating the effects of implementing sustainable initiatives on a firm's performance is an essential issue at present because of the rising concern about the depletion of resources and environmental conservation. While previous research considered green innovation practices, its application is dominantly observed in the manufacturing sector, with very few inclined towards adopting these practices in the transportation industry, which according to the International Energy Agency [40], is one of the most significant contributors of carbon emissions, accounting for 27% of the global emissions. Thus, the study focused on determining the critical elements of GII in the transportation industry in the Philippines and its effect on a firm's competitiveness and financial performance. The study considered the external and internal forces driving a firm's GII implementation. These include environmental regulations (ER), market demand (MD), government pressure (GP), and competitor pressure (CP) as the external elements, while corporate social responsibility (CSR) and employee conduct (EC) are the internal elements.

The study's theoretical model validated the relevance of implementing sustainable practices and their effect on an organization's competitive and financial performance. The findings presented in the study will aid the transportation sector in its decision-making activities. The study also aims to encourage the continuous implementation of green innovation initiatives to benefit the environment, society, and business.

The following is the study's organizational structure. First, the theoretical foundation is presented. The participants, survey, and model are then discussed in detail, along with the methodology. Next is the presentation of the results or outcomes. The final section of the study examines the main findings, the study's constraints, and its application. The study's key conclusions, limitations, and practical implications are discussed in the final section.

## 2 Literature review and conceptual framework

### 2.1 External and internal drivers of green innovation initiatives

Green innovation initiatives (GII) consider creating a product, process, or management strategy unique to the organization to reduce its environmental footprint and incorporate environmental advantages [41]. Its external and internal elements more often influence the adoption of GII by an organization. This study identified various drivers of GII, which include environmental regulations (ER), market demand (MD), government pressure (GP), competitor pressure (CP), corporate social responsibility (CSR), and employee conduct (EC).

Environmental regulation (ER) considers the administrative penalties imposed on companies that pollute the environment and the relevant laws and regulations enacted by the government [42]. Governments institute ERs to ensure that business owners will adhere to ecological conservation and protection, regardless of the type of business being operated. ERs positively affect green innovation initiatives [12,43] and are an external institutional driver that positively influences green process innovation and technological innovation [38]. The cost and penalty for violating ERs are driving forces to organizations since it entails financial implications and reputational damage. Peng et al. [39] also emphasized that incentive environmental regulation promotes green product innovation, process innovation, and terminal governance innovation, while command-and-control environmental regulation promotes green process innovation. Shan et al. [44] highlighted the role of green innovation and initiatives of renewable energy which, together with the transportation industry promoted an increase in carbon emission. This led to health hazards and waste. In addition, these problems are suggested to be addressed by Umar et al. [45]. To which, a highlight on the financial development and infrastructure in the transportation sector was considered in their study using wavelet coherence and cointegration approach. Most established theories have utilized mathematical model to demonstrate the coherence and relationship of financial and environmental issues raised in accordance with regulation and innovation initiatives among green manufacturing to cater to market demands.

Market demand (MD) enables companies to coordinate with their customers to know and observe their specifications and provide what they genuinely want in sustainable products to accomplish green process innovation [41,46]. Some coercive mechanisms that force businesses to adopt green initiatives include customer demands to set environmental standards, the provision of incentives, encouragement to adopt green practices, and customer rejection of products containing hazardous elements [47]. Various studies have indicated that market demand positively affects green innovation initiatives [12,27]. Ong et al. [48] identified how the transport or logistics sector rapidly impacts the environment, demonstrating how customers are becoming more conscious of their environmental obligations and pushing various transport industry stakeholders. The study of Irfan et al. [33] was inclusive of green finance in relation with green innovation and sustainable economic transformation. In relation, Asadi et al. [49] expounded on the market demand and opportunities brought by sustainability practices. Through structural equation modelling, their study developed a model covering strategy, culture, conservation, and regulations forming green innovation. This was further related to sustainable business performance. A positive significant effect was seen on social, environmental, and economic performances due to green innovation. It was explained that since the market are more aware of sustainable practices, their demand would be aligned with the need for consideration of GII among industries. Other studies [33,50] related these demands to cause great pressure among policymakers and highlighted comprehension among these parties for GII to be implemented.

Government pressure (GP) is critical to the success of a business, especially in international operations. Knowledge of international laws and local regulatory requirements enables business owners to create strategies to achieve global recognition [28]. Similarly, competitive pressure (CP) impacts a firm's innovation pace and scope [51]. It requires organizations to be accustomed to the industry standards and become aware of the competitors' products or services and actions [28]. Most studies have indicated that higher innovative adoption is related to higher competitive pressure [52]. In the manufacturing and service sectors, governmental and competitive forces were found to impact green innovation practices positively [28,53], influencing organizations to pay close attention to both international and domestic legislations to address the challenges of the global market [28].

Corporate social responsibility (CSR) and green organizational culture are well known for their essential contributions to organizational and societal growth [54]. CSR delivers competitive advantages and favorable economic benefits to companies as it emphasizes the significance of actively focusing on social and environmental elements of operations and stakeholder interaction [55]. Green management strategies are included in CSR practices for the company's many stakeholders [56]. Ren et al. [57] emphasized that green innovation performance is significantly better in companies with robust CSR implementation than in companies that do not report CSR. The findings of various studies also indicate that CSR promotes green processes and product innovation [55,36]. While CSR is commonly noted for civic or community engagement, organizations have also incorporated eco-friendly campaigns to promote sustainable practices.

Employee conduct (EC) embodies acceptable or professional behavior that shows consistent appreciation and consideration of environmental drivers to produce better performance and improved innovation [58]. Employee conduct and environmental concerns can lessen how negatively company actions affect the environment [59], while the top management is responsible for educating its employees on the practice of social and environmental responsiveness [60]. The study of Wang et al. [28] indicated that employee conduct positively impacts the green innovation practices of an organization. Similarly, a strong correlation exists between employees' green behavior and organizational environmental success [61]. Employee empowerment and engagement in sustainable activities are fostered through green cultures. It includes employee engagement in environment-friendly activities, contribution to improvement ideas, and employers' commitment to promoting sustainability through knowledge programs, training, and education.

### 2.2 Firm's competitiveness and financial performance

The study determined the significant influence of GII on a company's competitiveness and economic or financial performance. Firm competitiveness (FC) represents the enterprise's embodiment of higher-level global advantages and capital flow [62]. It forces firms to concentrate on their core competencies and outsource other crucial jobs due to the growing number of competitors in the business [63]. Higher green innovation performance from a worldwide standpoint aids business in enhancing their brand recognition and effectively managing overseas subsidiaries, which enhances overall performance [64]. Financial performance (FP) measures how well a company's strategy and operations are carried out, and it may fully capture the impact of cost management, asset management, funding allocation, and the make-up of shareholders' equity return rate [62]. Fiala and Hedija [65] identified that small businesses expand faster because they are more adaptable, have better access to the innovation process, or work harder to quickly reach an efficient production volume. Examining the influence of green initiatives on a company's competitiveness and financial performance is essential to determining the extent to which sustainable practices provide a competitive advantage and understanding the potential benefits and drawbacks of sustainable practices. Businesses can use it to find opportunities for cost savings, adhere to government regulations, attract new markets, and promote stakeholder engagement. By evaluating the effects of these initiatives, businesses may strategically coordinate their sustainability programs to produce economic success and achieve competitiveness in a constantly changing business environment. While organizations' efforts to innovate on a greener basis frequently face a financial challenge [66], various researchers emphasize that engaging in green industry practices improves reputation and financial performance [67,68]. Moreover, green processes and product innovation influence a firm's competitiveness and financial performance [62,69,70]. Supported by these studies, the study posited the following hypotheses:

H1 Green innovation initiatives (GII) have a significant positive relationship with a firm's competitiveness (FC).

H2 Green innovation initiatives (GII) have a significant positive relationship with a firm's financial performance (FP).

**Fig. 1:**
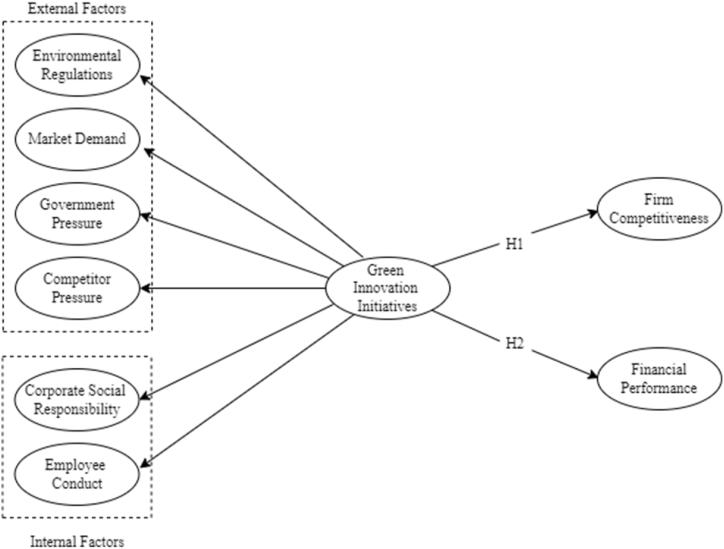
Conceptual framework.

Fig. 1 presents the conceptual framework. The study examined the impact of green innovation initiatives (GII) on a firm's competitiveness and financial performance in the transportation industry of the Philippines. GII is measured by the external and internal driving forces, which are environmental regulations (ER), market demand (MD), government pressure (GP), competitor pressure (CP), corporate social responsibility (CSR), and employee conduct (EC).

## 3 Methodology

### 3.1 Participants of the study

The study used a non-probability sampling technique called purposeful sampling, in which situations, individuals, or events are purposefully chosen to offer crucial information that cannot be acquired from other selections [71]. The respondents of this study were employees of companies in the Philippines engaged in the manufacture, distribution, retail, and service of motor vehicles. These include individuals from the rank and file (i.e., secretary, clerk, inspectors, serviceman, and others), low-level management (i.e., supervisor, line leader, and others), middle-level management (i.e., manager, assistant manager, director, department head, and others), and top-level management (i.e., president, CEO, VP, AVP, and others) to gather varying perspectives since all kinds of employees can significantly contribute to the practice and implementation of green innovation initiatives. A formal letter of request/endorsement was sent to the company's Human Resources Department, bearing the study's objectives and online survey link. The survey questionnaire was created online using Google Forms. Before the data collection, as indicated in the preface of the online survey, each participant gave their consent after being fully informed in compliance with the Data Privacy Act of the Philippines. The Mapua University Research Ethics Committees also approved this study (FM-RC-22-62). The study targeted 250 responses, but only 206, or 82.40%, were collected for two (2) months, from June 1 to July 31, 2022. Memon et al. [72] suggested that a sample of at least 160 valid observations is suitable for multivariate statistical analysis techniques such as CB-SEM and PLS-SEM, where it is not considered a small or large sample size. Using the Taro Yamane [73] equation, the samples collected demonstrated a remarkable representation of the total population with an error of 7%.

### 3.2 Questionnaire

The questionnaire contains four (4) sections. The first section pertains to the demographic profile of the respondents, which included sex, age, marital status, company location, job level at the firm, highest educational attainment, years of experience in the transportation industry, size of the organization, and the type of product or service offered. The second section corresponds to the elements of GII, which included six (6) variables and 19 items or constructs. The variables were environmental regulations (ER), market demand (MD), government pressure (GP), competitor pressure (CP), corporate social responsibility (CSR), and employee conduct (EC). The third section represents the firm's competitiveness (FC) and financial performance (FP) variables, where each considered five (5) items or constructs. The survey utilized a 5-point Likert scale ranging from one (1) as strongly disagree and five (5) as strongly agree.

### 3.3 Structural equation modelling (SEM)

The study utilized multivariate analysis using structural equation modeling (SEM) to examine the survey data. SEM measures the relationship of theoretical variables [63,74] and has the advantage of analyzing all pathways in a single analysis where the exploratory and dependent factors can be linked directly or indirectly [75]. This study applied the partial least square structural equation modeling (PLS-SEM), a type of analysis for creating constructs and testing the association between constructs by factoring in observed variables [76]. PLS-SEM is reliable in discovering optimum factors through factoring and amplifying the variables described by how much the construct impacts endogenous variables. Hair et al. [76] claimed that the (PLS-SEM) technique is better for complex and simple models. It is also useful when executing estimations and is superior to regression in measuring mediation [77].

### 3.4 Hierarchical latent variable model

Hierarchical latent variable models, also known as higher-order constructs, are distinguished based on the number of levels in the model, which are composed of second-order models [78] that illustrates the relationships between constructs [79]. This model type reduces the number of relationships between path models [80]. The study demonstrates higher-order reflective constructs as it measures and describes the unobservable variables of GII [81], which are environmental regulations (ER), market demand (MD), government pressure (GP), competitor pressure (CP), corporate social responsibility (CSR), and employee conduct (EC). The model has three (3) latent variables, which are green innovation initiatives (GII), firm's competitiveness (FC), and financial performance (FP), and fifteen (15) constructs.

## 4 Results

### 4.1 Data analysis

Table 1 shows the profile of the participants. A total of 206 employees of motor vehicle companies responded to the survey. Regarding gender, 61.17% are female, and 38.83% are male. When it comes to the age of the respondents, 14.56% are between 18 and 25 years old, 48.06% are between 26 and 35 years old, 21.36% are between 36 and 45 years old, 11.65% are between 46 and 55 years old, while 4.37% are above 55 years old. Regarding marital status, 58.25% of the respondents are single, and 38.83% are married. In contrast, 2.91% are neither single nor married. For the company location, 88.83% of the respondents work in an urban area, while 11.17% work in a rural area. Regarding the respondents' job level at the firm, 1.94% belonged to top-level management, 14.56% were from middle-level management, 25.24% were from lower-level management, and 58.25% were rank-and-file employees. For the respondents' educational attainment, 3.88% finished high school, 13.59% finished vocational, 80.10% obtained a bachelor's degree, 1.94% obtained a master's degree, and 0.49% obtained a doctorate. Regarding the respondents' years in the industry, 14.08% have less than one year of experience, 20.39% have 1–3 years of experience, 27.67% have 4–6 years of experience, 12.14% have 7–10 years of experience, and 25.73% have more than ten years of experience. On the size of the organization or enterprise where the respondents work, determined by the employment and asset size, 6.31% were employed in a small organization, 21.36% were from a medium organization, and 72.33% were working in a large organization. A small enterprise employs 10 to 99 employees and has an asset size of Php 3,000,001 to Php 15,000,000, a medium with 100 to 199 employees and an asset size of Php 15,000,001 to Php 100,000,000, and a large has 200 or more employees and an asset size of more than Php 100,000,000 [82]. For the type of motor vehicle manufactured, sold, or serviced, 2.91% for motorcycles and tricycles, 91.26% for private vehicles, and 5.83% for public vehicles.

**Table 1:** Respondents’ profile (*n = 206*).

Criteria	Category	N	%
Sex	Female	126	61.17%
	Male	80	38.83%
	*Total*	*206*	*100.00%*
Age	18–25	30	14.56%
	26–35	99	48.06%
	36–45	44	21.36%
	46–55	24	11.65%
	Above 55	9	4.37%
	*Total*	*206*	*100.00%*
Marital Status	Single	120	58.25%
	Married	80	38.83%
	Others	6	2.91%
	*Total*	*206*	*100.00%*
Company Location	Urban	183	88.83%
	Rural	23	11.17%
	*Total*	*206*	*100.00%*
Job level at the firm	Top-level management	4	1.94%
	Middle-level management	30	14.56%
	Low-level management	52	25.24%
	Rank and file	120	58.25%
	*Total*	*206*	*100.00%*
Educational Attainment	Highschool	8	3.88%
	Vocational	28	13.59%
	Bachelor's Degree	165	80.10%
	Master's Degree	4	1.94%
	Doctorate Degree	1	0.49%
	*Total*	*206*	*100.00%*
Years experienced in the industry	Less than 1 year	29	14.08%
	1–3 years	42	20.39%
	4–6 years	57	27.67%
	7–10 years	25	12.14%
	More than 10 years	53	25.73%
	*Total*	*206*	*100.00%*
Size of Organization/Enterprise	Small	13	6.31%
	Medium	44	21.36%
	Large	149	72.33%
	*Total*	*206*	*100.00%*
Type of Product/Service Offered	Motorcycles and Tricycles	6	2.91%
	Private Vehicles (cars, SUVs, etc.)	188	91.26%
	Public Vehicles (jeepney, bus, truck, etc.)	12	5.83%
	*Total*	*206*	*100.00%*

Cronbach's α, composite reliability (CR), and average variance extracted (AVE) were used to test the model. Cronbach's α was applied since it is a commonly used indicator for measuring the internal consistency of multiple-item measurement instruments [83]. Composite reliability was utilized to measure the reliability of a particular construct through item loadings [84]. According to Hair et al. [85], the construct scores of Cronbach's α and composite reliability should be ≥ 0.70 to be considered acceptable on both tests. These two tests must be reported to demonstrate the dependability of weighted and unweighted measures [86]. The AVE represents the amount of variance in the indicators that can be accounted for by the underlying latent variable, where an AVE value to be considered a good measurement should be ≥ 0.50 [84,87]. Lastly, the indicator reliability for the outer loadings should have values ≥ 0.70 to be considered acceptable [76].

**Fig. 2:**
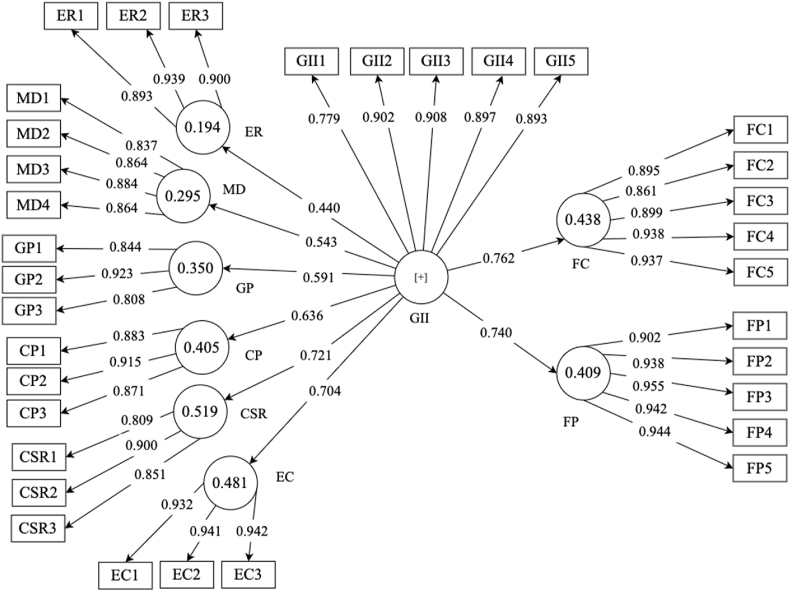
Measurement model.

The summary of measurements of the first and second order constructs are shown in Table 2, Table 3, respectively. The Cronbach's alpha values greater than 0.70 indicate that the results are acceptable [87]. Further, measurements of the CR and AVE values are deemed reliable and valid because it falls within the threshold range [76]. Fig. 2 exhibits the measurement model, which shows that the values of the outer loadings are considered satisfactory since they are greater than 0.70 [76]. The Harman's single factor test was also used and yielded a value of 32.80% for the common method bias. This value is lower than 50%, the acceptable common method bias threshold [76]. As a result, there was no common method bias. Using SPSS 25, the Shapiro-Wilk statistical test was performed, and the results indicated that the data is normally distributed since both skewness and kurtosis quotient values were within a 1.96 range.

**Table 2:** Measurement of first-order constructs.

Variables	Items	Mean	Loadings (≥0.70)	Cronbach's Alpha (≥0.70)	Composite Reliability (≥0.70)	Average Variance Extracted (≥0.50)
Green Innovation Initiatives	GII1	3.816	0.779	0.924	0.943	0.770
	GII2	3.816	0.902			
	GII3	3.913	0.908			
	GII4	3.903	0.897			
	GII5	3.845	0.893			
Firm Competitiveness	FC1	3.714	0.895	0.946	0.958	0.822
	FC2	3.762	0.861			
	FC3	3.723	0.899			
	FC4	3.650	0.938			
	FC5	3.680	0.937			
Financial Performance	FP1	3.655	0.902	0.965	0.973	0.877
	FP2	3.626	0.938			
	FP3	3.631	0.955			
	FP4	3.592	0.942			
	FP5	3.607	0.944

**Table 3:** Measurement of second-order constructs.

Variables	Items	Mean	Loadings (≥0.70)	Cronbach's Alpha (≥0.70)	Composite Reliability (≥0.70)	Average Variance Extracted (≥0.50)
Environmental Regulations	ER1	3.864	0.893	0.898	0.936	0.830
	ER2	3.762	0.939			
	ER3	3.748	0.900			
Market Demand	MD1	3.782	0.837	0.886	0.921	0.744
	MD2	3.621	0.864			
	MD3	3.602	0.884			
	MD4	3.699	0.864			
Government Pressure	GP1	3.650	0.844	0.823	0.895	0.740
	GP2	3.680	0.923			
	GP3	3.850	0.808			
Competitor Pressure	CP1	3.864	0.883	0.869	0.919	0.791
	CP2	3.796	0.915			
	CP3	3.800	0.871			
Corporate Social Responsibility	CSR1	4.053	0.809	0.814	0.890	0.730
	CSR2	3.665	0.900			
	CSR3	3.549	0.851			
Employee Conduct	EC1	3.777	0.932	0.932	0.957	0.880
	EC2	3.728	0.941			
	EC3	3.811	0.942

### 4.2 Structural Model Assessment

**Fig. 3:**
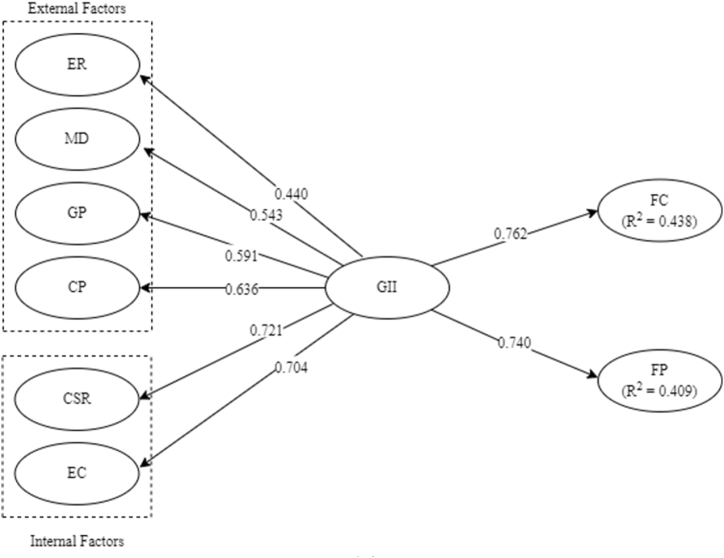
Model assessment.

In the structural path analysis shown in Table 4 and Fig. 3, only direct effects were present and no indirect effects. The direct effect values obtained for the paths of GII to FC and FP are 0.762 and 0.740, respectively.

**Table 4:** Structural path analysis.

Paths		Direct Effect (β)
1	GII → FC	0.762
2	GII → FP	0.740

Table 5 presents the result of hypothesis validation. It can be observed that both hypotheses H1 and H2 are accepted since the p-values are within the threshold. Green innovation initiatives demonstrated a significant positive relationship to both firm competitiveness (*β* = 0.762, *p* < 0.01) and financial performance (*β* = 0.740, *p* < 0.01).

**Table 5:** Hypothesis validation.

Paths		*p*-values	Decision
H1:	GII → FC	0.000	Accepted
H2:	GII → FP	0.000	Accepted

The results of the model fit tests are summarized in Table 6. The variance inflation factor (VIF) was used to evaluate the degree of collinearity in PLS-SEM, where values greater than five (5) indicate a potential issue with the collinearity problem [88]. The highest VIF value among the variables utilized in this study is 4.081, proving no collinearity between the variables. A value smaller than 0.08 for the standardized root mean squared residual (SRMR) denotes a better model fit. The obtained SRMR value of 0.048 is also within the threshold [89]. The Chi-square upon the degree of freedom with a value of 3.45 denotes the adequacy of the overall model fit [90]. The normed fit index (NFI) of 0.923 exceeds the threshold of 0.90, indicating that the model is well-fitted [91].

**Table 6:** Model fit result.

Parameters	Values	Threshold	References
Variance Inflation Factor (VIF)	1.605 to 4.081	≤5.00	[79]
Standardized Root Mean Squared Residual (SRMR)	0.048	≤0.08	[80]
Chi square/df	3.450	≤5.00	[81]
Normed Fit Index (NFI)	0.923	≥0.90	[82]

To assess the discriminant validity, the Fornell-Larcker Criterion (FLC) and Heterotrait-Monotrait Ratio (HTMT) were utilized. FLC is a conventional approach to evaluate the constructs' correlation with the square root of the average variance extracted (AVE) for each latent variable [92]. As observed in Table 7, the diagonal values for each variable or factor are higher than the correlation value between the latent variables in their respective horizontal values, which means these parameters are satisfactory [93]. The Heterotrait-Monotrait Ratio (HTMT) is another method utilized to test the discriminant validity to provide a different stance or viewpoint. Discriminant validity examines the overall fit of the constructs of the model. According to Hair et al. [76], the value recommended for Heterotrait-Monotrait Ratio (HTMT) to be deemed acceptable should be ≤ 0.900. The results of the HTMT shown in Table 8 indicate that all constructs in the study are acceptable.

**Table 7:** Fornell-Lacker criterion (FLC).

Factors	CP	CSR	EC	ER	FC	FP	GII	GP	MD
CP	**0.890**								
CSR	0.616	**0.854**							
EC	0.614	0.689	**0.938**						
ER	0.546	0.424	0.445	**0.911**					
FC	0.591	0.569	0.623	0.451	**0.907**				
FP	0.588	0.570	0.645	0.429	0.753	**0.936**			
GII	0.636	0.721	0.694	0.440	0.662	0.640	**0.877**		
GP	0.626	0.571	0.578	0.663	0.563	0.579	0.591	**0.860**	
MD	0.649	0.586	0.603	0.698	0.576	0.583	0.543	0.622	**0.862**

**Table 8:** Heterotrait-Monotrait Ratio (HTMT).

Factors	CP	CSR	EC	ER	FC	FP	GII	GP
CSR	0.736							
EC	0.682	0.792						
ER	0.617	0.496	0.485					
FC	0.651	0.650	0.663	0.484				
FP	0.641	0.644	0.679	0.460	0.789			
GII	0.701	0.831	0.744	0.478	0.707	0.675		
GP	0.741	0.698	0.657	0.771	0.640	0.652	0.674	
MD	0.737	0.688	0.661	0.787	0.629	0.634	0.591	0.728

## 5 Discussion

The study conveys significant positive correlations of green innovation initiatives to the firm's competitiveness and financial performance.

The first notable finding of the study is the significant influence of environmental regulations (ER), market demand (MD), government pressure (GP), competitor pressure (CP), corporate social responsibility (CSR), and employee conduct (EC) to the implementation of green innovation initiatives in the transportation sector. CSR (β = 0.721) and EC (β = 0.704), considered internal drivers, were the most substantial in applying GII. This implies that motor vehicle manufacturers, retailers, and service providers in the Philippines prioritize customer satisfaction through manufacturing, selling, and using green products. They also recognize that providing financial aid to underprivileged groups in society is an essential component of their corporate mission. This result is supported by various literature [36,55] which indicated that CSR significantly influences green innovation. Similarly, the company's culture, through the employees' attitudes and behavior, is also a key to the success of the organization's green practice. It is evident that if the workforce does not support the policies and strategies of the organization, then the organization will struggle to fulfil its environmental goals [94]. Employee knowledge and awareness of the GII as well as the encouragement and commitment of the top management in promoting the initiatives, are also vital to its successful implementation. This finding is supported by the study of Wang et al. [28], which indicates that EC significantly influences green innovation in manufacturing and service firms. Among the external drivers, CP (β = 0.636) was found to be the most dominant. Organizations adhere to local and global environmental standards to be at par with their competitors and maintain a good reputation for conserving the environment. Maintaining a good company image is a commitment to stay in the market and be recognized. This finding is supported by various researchers who identified that competitive forces positively influence green innovation practices [28,53].

The second remarkable finding in this study is the strong positive influence of GII on the firm's competitiveness (β = 0.762, p < 0.01). Implementing green practices not only positively enhances the image and reputation of the company but also helps gain the trust and support of the current and future market. This support enables the company to attract investments in the process, further strengthening a firm's competitiveness and ultimately contributing to better financial performance [28,95]. A firm can enhance competitiveness by demonstrating a friendly image, increasing market share, investing in research and development, and augmenting customer satisfaction. According to the correlation statistics, there is a significant association with a value of 0.438 since an R^2^ value greater than 0.10 is deemed substantial [96]. The finding is supported by various researchers who identified that GII positively influences a firm's competitiveness [28,64,67,68,70,95].

The third significant finding of the study is the strong positive relationship of GII to the firm's financial performance (β = 0.740, p < 0.01). A company implementing green innovation initiatives attracts more customers and environmentally conscious individuals and regards companies participating in environmental conservation programs and activities [63]. Furthermore, Nadlifatin et al. [97] found that businesses that sell green products have favorable effects with minimal effort. Xie et al. [69] also recognized that although the initial costs of deploying new environmental technologies are often higher, a positive association between green innovations and financial performance will emerge over time. Additionally, according to Benkraiem et al. [98], reducing emissions over time benefits businesses financially, and corporate investors offer financial incentive to support green innovation. The R^2^ value of 0.409 demonstrates a meaningful relationship between variables [99]. This finding is supported by various studies which specified that implementing green industry practices improves the financial performance of the company [62,[67], [68], [69], [70]].

Generally, the study demonstrated several external and internal drivers that are significant to the practice of green innovation initiatives in the transportation sector in the Philippines. These driving forces are environmental regulations, market demand, government pressure, competitor pressure, corporate social responsibility, and employee conduct. Consequently, implementing green innovation initiatives positively influences the firm's competitiveness and financial performance. Companies that adopt sustainable projects are optimistically rewarded in several ways, such as growing market share, enhancing competitive advantage, and receiving financial recognition.

### 5.1 Managerial implications

The study's findings highlighted the role of corporate social responsibility and employee conduct in successfully implementing green innovation initiatives. Organizations at present should integrate the concept of sustainability into their CSR practices and ensure the participation and commitment of every employee. Adapting a green culture in the organization will benefit society and the environment. The management may provide rewards or incentives to employees to motivate them to be committed to the effective practice of green initiatives in the workplace. These incentives can be given as monetary, material, or other non-financial incentives.

The second recommendation presents the positive impact of green innovation initiatives on the firm's competitiveness and financial performance. Companies can highlight their green innovation initiatives as a marketing strategy to be recognized and gain more exposure in the market, ultimately informing a broader public audience or potential customers. Similarly, since customers are more inclined to consume green products and services, such an initiative will attract them and potentially increase sales and profit. Another economic benefit of green practice is the savings that can be generated from reducing carbon footprint in workstations. Such savings may be spent on sustainable research and development projects such as renewable energy, electric motor vehicles, and others. Several potential mechanisms may be considered for the promotion of GII and financial performance.

One aspect a company may look at may be cost reduction and market differentiation. Several strategies may be considered for the development which may affect the perception of stakeholders. By focusing on less energy usage, developed technologies, and fewer materials, both companies and industries can reduce costs. An advantage may also be considered due to less energy usage and promoting sustainable practices which consumers are more likely to consider. Suggestions such as focusing on simplified packaging may reduce costs and promote effective environmental support, even though recycling. Transitioning to sustainable practices and green manufacturing may be practiced on a small scale which will gain financial improvements. As mentioned in the study of Tjahjadi et al. [100], these practices were shown to have a significant effect on green market orientation and business performances. They have presented a positive influence on market segmentation and market differentiation of green practices among small and medium enterprises. The balance on societal, economic, and environmental practices should be focused on better market differentiation. The innovation aspects focusing on green technologies prompts a positive response among the community, which should be seen on the practices of these industries. As explained in the study of Ong et al. [48], the development of practices may be a first step which consumers would continuously support until the end cycle. Therefore, stakeholders and industries can establish these relationships and expose the green practices for better community development and interaction – leading to better financial performance.

### 5.2 Limitations

The present research yielded informative results concerning implementing green innovation initiatives in the transport sector. However, several limitations must be addressed in future studies. First, the study focused only on employees of land motor vehicle companies engaged in the manufacture, distribution, retail, and service. The study applies to other transportation sectors, such as maritime, aviation, and railway. Other industries in the Philippines and in other countries may also be considered to gain a global perspective on the implementation of green innovation initiatives. Second, the data was collected over two (2) months with 206 respondents. Increasing the number of respondents will increase data accuracy and reliability. Finally, the study considered only six (6) drivers of green innovation initiatives. Future studies may include other drivers or elements of green innovation initiatives, such as innovation cost, social influence, management's commitment, and other factors.

## 6 Conclusion

The study determined the significant influence of green innovation initiatives on motor vehicle firms' competitiveness and financial performance in the Philippines. The initiatives were driven by external and internal factors such as environmental regulations, market demand, government pressure, competitor pressure, corporate social responsibility, and employee conduct. The results indicated that the six (6) driving forces are essential to implementing the initiatives, with corporate social responsibility and employee conduct as the most relevant since the people in the organization are the primary actors or implementors of a company's sustainable practices. Organizations are encouraged to incorporate not only a concern for society or community but also a concern for the environment as part of their CSR. Further, to encourage and motivate the workers to adhere to green practices, rewards and incentives in various forms may be given to them. The study also presented the positive effect of green innovation initiatives on a firm's competitiveness and financial performance. Organizations with sustainable practices acquire better market leverage and position because consumers are interested in products or services that protect the environment. This interest encourages loyalty and continued patronage that, in effect, help organizations gain more income. Sustainable practices also result in cost reduction, which makes organizations financially competitive. The implementation of green innovation initiatives should be demonstrated by motor vehicle companies and across all industries.

## Production notes

### Author contribution statement

Josephine D. German, Anak Agung Ngurah Perwira Redi, Ardvin Kester S. Ong, and Jerome L. Liwanag: Conceived and designed the experiments; Performed the experiments; Analyzed and interpreted the data; Contributed reagents, materials, analysis tools or data; Wrote the paper.

### Data availability statement

Data will be made available on request.

## Declaration of competing interest

The authors declare that they have no known competing financial interests or personal relationships that could have appeared to influence the work reported in this paper.
