# Supplementary material for: The impact of green innovation initiatives on competitiveness and financial performance of the land transport industry
Source: Heliyon. 2023 Aug 15;9(8):e19130. doi: 10.1016/j.heliyon.2023.e19130 (PMC10457538; doi:10.1016/j.heliyon.2023.e19130)
Supplement: Multimedia component 1 [file mmc1.docx]

**Questionnaire**

| Variable | Code | Description | References |
| --- | --- | --- | --- |
| Environmental Regulations | ER1 | The government has created environmental regulations for the transportation industry. | Raza, (2020); Yang, (2018); Zhu et al. (2013) |
|  |  |  |  |
|  |  |  |  |
|  | ER2 | There are clear requirements for the pollutant emission intensity values for the transportation industry. | Peng et al. (2021) |
|  |  |  |  |
|  |  |  |  |
|  | ER3 | Tradable permits and pollution control subsidies play a role in stimulating innovation enthusiasm in the transportation industry. | Peng et al. (2021) |
|  |  |  |  |
|  |  |  |  |
|  |  |  |  |
| Market Demand | MD1 | There is a segmentation of the market for the transportation industry. | Lin et al. (2013); Oltra and Jean, (2009); Kammerer, (2009) |
|  |  |  |  |
|  | MD2 | Customer requirements about green products are met or satisfied. | Lin et al. (2013); Oltra and Jean, (2009); Kammerer, (2009) |
|  |  |  |  |
|  |  |  |  |
|  | MD3 | Price flexibility of demand for green products is always occurring. | Lin et al. (2013); Oltra and Jean, (2009); Kammerer, (2009) |
|  |  |  |  |
|  |  |  |  |
|  | MD4 | Customer benefit for green products is high. | Lin et al. (2013); Oltra and Jean, (2009); Kammerer, (2009) |
|  |  |  |  |
| Government Pressure | GP1 | Regulation for green products in our country is stringent. | Wang et al. (2021a) |
|  |  |  |  |
|  | GP2 | Future regulation for green products in our country is predictable. | Wang et al. (2021a) |
|  |  |  |  |
|  | GP3 | Regulations for green products have a considerable impact on business entities in the country. | Wang et al. (2021a) |
|  |  |  |  |
|  |  |  |  |
| Competitor Pressure | CP1 | Our major competitors practice worldwide environmental standards for their operations and products. | Wang et al. (2021a) |
|  |  |  |  |
|  |  |  |  |
|  | CP2 | Our major competitors implement environmental strategies on a worldwide basis. | Wang et al. (2021a) |
|  |  |  |  |
|  |  |  |  |
|  | CP3 | Implementing an environmental strategy can affect considerably our environmental reputation with competitors in other countries. | Wang et al. (2021a) |
|  |  |  |  |
|  |  |  |  |
| Corporate Social Responsibility | CSR1 | We use customer satisfaction as an indicator of whether to continue producing/using green products. | Yuan and Cao, (2022) |
|  |  |  |  |
|  |  |  |  |
|  |  |  |  |
|  | CSR2 | We set aside an amount from our budget for green transportation projects. | Yuan and Cao, (2022) |
|  |  |  |  |
|  |  |  |  |
|  | CSR3 | We set aside an amount from our budget for providing for the underprivileged groups in society. | Yuan and Cao, (2022) |
|  |  |  |  |
|  |  |  |  |
|  |  |  |  |
| Employee Conduct | EC1 | The top management's behavior inspired the acceptance of change by all the other organization members. | Wang et al. (2021a) |
|  |  |  |  |
|  |  |  |  |
|  | EC2 | The employees were able to take initiative and decisions on their own thanks to the encouragement of authority delegation. | Wang et al. (2021a) |
|  |  |  |  |
|  |  |  |  |
|  | EC3 | All the organization members knew and shared the firm's mission and objectives. | Wang et al. (2021a) |
|  |  |  |  |
| Green Innovation Initiatives | GII1 | We consistently recycle, reuse, and remanufacture materials or parts. | Wang et al. (2021b) |
|  | GII2 | We redesign our production and operation processes to improve environmental efficiency. | Wang et al. (2021b) |
|  |  |  |  |
|  |  |  |  |
|  |  |  |  |
|  | GII3 | We redesign and improve products or services to meet new environmental criteria or directives. | Wang et al. (2021b) |
|  |  |  |  |
|  |  |  |  |
|  | GII4 | We use less or non-polluting/toxic materials that are environmentally friendly. | Wang et al. (2021b) |
|  |  |  |  |
|  |  |  |  |
|  | GII5 | We use eco-labelling in our products or materials/parts. | Wang et al. (2021b) |
|  |  |  |  |
| Firm Competitiveness | FC1 | Our environment-friendly image in the transportation industry is higher than our competitors. | Wang et al. (2021b); Dong et al. (2014); Huang et al. (2016); Sellitto et al. (2020) |
|  |  |  |  |
|  |  |  |  |
|  |  |  |  |
|  | FC2 | Our overall market competition advantage is greater than our competitors. | Wang et al. (2021b); Dong et al. (2014); Huang et al. (2016); Sellitto et al. (2020) |
|  |  |  |  |
|  |  |  |  |
|  | FC3 | Our market share of product/service increased faster than our competitors. | Wang et al. (2021b); Dong et al. (2014); Huang et al. (2016); Sellitto et al. (2020) |
|  |  |  |  |
|  |  |  |  |
|  | FC4 | Our proportion of research and development investment is higher than our competitors. | Wang et al. (2021b); Dong et al. (2014); Huang et al. (2016); Sellitto et al. (2020) |
|  |  |  |  |
|  |  |  |  |
|  | FC5 | Our consumer satisfaction with products/services is higher than our competitors. | Wang et al. (2021b); Dong et al. (2014); Huang et al. (2016); Sellitto et al. (2020) |
|  |  |  |  |
|  |  |  |  |
| Financial Performance | FP1 | Our profitability of new products/services reached the leading level of the industry when performing green innovation initiatives. | Wang et al. (2021b); Ma et al. (2021); Yook et al. (2018); Dong et al. (2014) |
|  |  |  |  |
|  |  |  |  |
|  |  |  |  |
|  | FP2 | New product/service sales increased as a percentage of total sales when performing green innovation initiatives. | Wang et al. (2021b); Ma et al. (2021); Yook et al. (2018); Dong et al. (2014) |
|  |  |  |  |
|  |  |  |  |
|  | FP3 | Our profitability improved faster than its competitors when performing green innovation initiatives. | Wang et al. (2021b); Ma et al. (2021); Yook et al. (2018); Dong et al. (2014) |
|  |  |  |  |
|  |  |  |  |
|  |  |  |  |
|  | FP4 | Our return on asset (ROA) improved faster than its competitors when performing green innovation initiatives. | Wang et al. (2021b); Ma et al. (2021); Yook et al. (2018); Dong et al. (2014) |
|  |  |  |  |
|  |  |  |  |
|  |  |  |  |
|  | FP5 | Our productivity improved faster than its competitors when performing green innovation initiatives. | Wang et al. (2021b); Ma et al. (2021); Yook et al. (2018); Dong et al. (2014) |
|  |  |  |  |
